# Heterogeneous Sono-Fenton like catalytic degradation of metronidazole by Fe_3_O_4_@HZSM-5 magnetite nanocomposite

**DOI:** 10.1016/j.heliyon.2023.e16461

**Published:** 2023-05-26

**Authors:** Ghazal Yazdanpanah, Mohammad Reza Heidari, Najmeh Amirmahani, Alireza Nasiri

**Affiliations:** a Environmental Health Engineering Research Center, Kerman University of Medical Sciences, Kerman, Iran; b Environmental Health Engineering, Department of Environmental Health, School of Public Health, Bam University of Medical Sciences, Bam, Iran

**Keywords:** Heterogeneous catalyst, Sono-Fenton like, Metronidazole, Wastewater

## Abstract

In this research, Fe_3_O_4_@HZSM-5 magnetic nanocomposite was synthesized via a coprecipitation method for metronidazole (MNZ) degradation from aqueous solutions under ultrasonic irradiation which showed superb sonocatalytic activity. The synthesized magnetite nanocomposite was characterized by using field-emission scanning electron microscope-energy dispersive X-ray Spectroscopy, (FESEM-EDS), Line Scan, Dot Mapping, X-ray diffraction (XRD), vibrating sample magnetometer (VSM), and Brunauer-Emmett-Teller (BET). To investigate the sonocatalytic activity of the Fe_3_O_4_@HZSM-5 magnetite nanocomposite, the sonocatalytic removal conditions were optimized by evaluating the influences of operating parameters like the dosage of catalyst, reaction time, pH, the concentration of H_2_O_2_, MNZ concentration, and pH on the MNZ removal. The MNZ maximum removal efficiency and TOC at reaction time 40 min, catalyst dose 0.4 g/L, H_2_O_2_ concentration 1 mM, MNZ initial concentration 25 mg/L, and pH 7 were achieved at 98% and 81%, respectively. Additionally, the MNZ removal efficiency in the real wastewater sample under optimal conditions was obtained at 83%. The achieved results showed that using Langmuir-Hinshelwood kinetic model K_L-H_ = 0.40 L mg^−1^, K_C_ = 1.38 mg/L min) can describe the kinetic removal of the process. The radical scavenger tests indicated that the major reactive oxygen species were formed by hydroxyl radicals in the Sono-Fenton-like process. Evaluation of the nanocomposite reusability showed an 85% reduction in the MNZ removal efficiency after seven cycles. Based on the results, it can be concluded that Fe_3_O_4_@HZSM-5 were synthesized as magnetic heterogeneous nano-catalysts to effectively degrade MNZ, and the observed stability and recyclability demonstrated that Fe_3_O_4_@HZSM-5 was promising for the treatment of wastewater contaminated with antibiotics.

## 1 Introduction

Hospital wastewater is one of the most infectious and dangerous wastewaters which may contain a large number of pathogenic microorganisms and dangerous contaminants such as antibiotics, drugs, and various hormones [1]. In recent years, concerns have been raised about the presence of a wide range of pharmaceutical materials in aquatic environments. Today, the use of antibiotics to improve human and animal health has received much attention [2]. Antibiotics are stable and lipophilic. They can maintain their chemical structure in the body for a long time, but these compounds are absorbed in small amounts by the body. A significant proportion of antibiotics enter the receiving waters through urine, stool, and hospital wastewater. Previous studies have shown that the concentration of antibiotics in hospital wastewater is in the range of 0 to 200 mg/L [3].

Metronidazole is among the most widely used antibiotics in the world, which has anti-inflammatory and antibacterial capabilities. It is used to treat infections as a result of anaerobic bacteria and protozoa. This chemical is the only medicine of the nitroimidazole group that has been included in the list of essential medicines by the World Health Organization (WHO) [4].

Physical, biological, and chemical methods are used in wastewater treatment plants to remove pollution from domestic and industrial wastewater [[5], [6], [7], [8], [9], [10], [11], [12], [13], [14], [15], [16], [17], [18]]. Nevertheless, these methods are not effective enough to remove medicine contaminants such as antibiotics [19]. Since the treatment systems type and antibiotics chemical structure have important roles in the removal rate of antibiotics, many researchers, in the previous decades, carried out a lot of studies to remove non-biodegradable antibiotics by the advanced oxidation processes (AOPs) from aqueous solutions [[20], [21], [22], [23]]. Fenton as an effective and feasible advanced treatment process has been advised for wastewater remediation [24]. The basis of these processes is the active radicals' formation under the acidic condition that reacts with the resistance pollutants and organic compounds. Therefore, their high oxidation capacity and non-selective activity can degrade all kinds of resistance pollutants. Active radicals such as hydroxyl, sulfate, superoxide, and hydroperoxyl are strong oxidants with a high tendency to destroy antibiotics [25]. The most important features of this technology are the high efficiency, low start-up and operation costs, and variety in methods used [26].

It should be noted that the Fenton process has many significant downside problems that limit its use on large scales. Of drawbacks of this process are chemical sludge production, low revival rate of ferric iron to ferrous iron, and decreased decomposition rate of H_2_O_2_ and, poor recycling of the homogenous catalyst. These problems can lead to an increase in the operational costs and process time [27]. Other kinds of heterogeneous catalysts such nano zero-valent iron and Fe^3+^ instead of Fe^2+^ can be used to overthrow the mentioned problems [28]. These kinds of chemical mechanisms are Fenton-like processes. A solid catalyst such as Fe_3_O_4_ magnetic nanoparticles (MNPs) because of their high catalytic activity has been used in the heterogeneous Fenton-like process [29]. In order to produce hydroxyl radicals and degrade organic pollutants, H_2_O_2_ molecules are broken in the presence of MNPs while a large amount of them remain in the solution in the solid phase and can reuse again [30]. In addition to Fe_3_O_4_ nanoparticles, other nanocomposites of this group have been used to improve the catalytic performance and efficiency of the Fenton process [31]. In AOPs processes, the minerals compounds such as kaolin [32], zeolite [33], and bentonite [34] which are composited with Fe_3_O_4_ nanoparticles have been used as catalysts.

Zeolites are a kind of minerals owned by the crystalline aluminosilicates class [33]. Due to their high specific surface area, pore structure, excellent ion-exchange performance, controllable acidity, high mechanical strength, high thermal stability, high porosity, and low-cost reactivation, zeolites are used as a popular and effective adsorbent to remove pollutants [35]. The application of chemical or thermal methods to modify the molecular sieves is useful to improve the properties of adsorbents and increase the removal process efficiency. Therefore, after the combination of zeolite with other catalyst metals of metal oxide materials, better removal is seen in the treatment processes [36].

Zeolite Socony Mobil-5 (ZSM-5) is an aluminosilicate zeolite with the H form or protonic type hydrogen that has Mordenite Framework Inverted (MFI)-type structure. This zeolite is frequently used as a supportive part for many heterogeneous catalysts to more effectively treat water and wastewater [37].

Today, the use of oxidizing agents such as hydrogen peroxide, persulfate, periodate, etc. to increase the performance of advanced chemical oxidation processes (AOP) with the aim of removing more organic pollutants has been considered by researchers [[38], [39], [40], [41], [42], [43], [44]]. In this method, an activating source such as ultraviolet (UV) [45] or ultrasound (US) is used [12].

Ultrasound is a wave with a frequency exceeding the human auditory capacity (20 to 40 kHz) that due to some ascendancies like high efficiency and not producing secondary pollutants in the environment has been used as an antimicrobial agent. Cavities creation or micro-bubbles resulting from the ultrasound cavitation in the water, which leads to the formation of the hole in the water that will eventually generate the hydroxyl radicals is the main mechanism of this process to oxidation of the pollutant. Applying the ultrasound waves along with catalysts to decompose the resistant and hazardous organic pollutants are taken into consideration [46]. The sound waves with a frequency higher than 16–20 kHz are used in the chemical reactions. According to Eqs. [1,2], ultrasound waves with the formation, growth, and destruction of holes in the liquid phase could lead to producing a lot of energy in the reactor [47].

**Figure:**
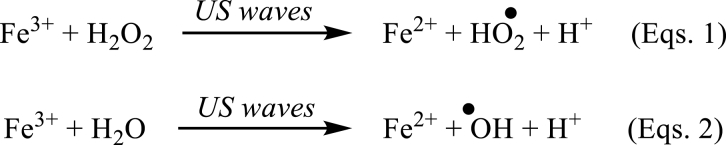

In this process, the energy of ultrasound waves is used to produce hydroxyl radicals as an active oxidizing agent as well as the oxidation of the organic compounds. In addition, the produced H_2_O_2_ because of sonolysis of water along with the presence of homogeneous and heterogeneous catalysts increases the decomposition of the organic matter. The decomposition rate of organic compounds using ultrasound waves is very low, therefore, various methods such as applying the heterogeneous iron oxide nanocatalysts are used to improve their efficiency [48]. Ferrite heterogeneous nanocatalysts such as CoFe_2_O_4,_ ZnFe_2_O_4_, Fe_3_O_4_, and CuFe_2_O_4_ have been used to remove organic and inorganic contaminants during the advanced oxidation processes [49].

The Sono-Fenton-like process is widely used to increase the organic pollutants degradation which involves the combination of a Fenton-like process and ultrasound irradiation [50]. Nonetheless, the behavior of this process and its by-products has not been studied real in wastewater until now. With the aim of degradation of metronidazole with a Heterogeneous Sono-Fenton-like method in the presence of Fe_3_O_4_@HZSM-5 magnetite nanocomposite, this catalyst was synthesized and characterized. Then, the effect of experimental parameters including pH, H_2_O_2_ concentration, initial metronidazole concentration, catalyst dosage, and ultrasonic power effect on the metronidazole removal efficiency was investigated. Besides, the function of Fe_3_O_4_@HZSM-5 magnetite nanocomposite catalyst was also studied on real wastewater.

## 2 Materials and methods

### 2.1 Chemical materials

The required chemical materials including metronidazole (with a purity of 9.99%), iron chloride (II), iron chloride (III), ammonia (NH_3_), and hydrochloric acid were purchased from Merck Company (Germany). Additionally, metronidazole (Microanalysis) (MNZ), and zeolite (HZSM-5) were prepared from DarouPakhsh Company and Iran Zeolite Company (Iran), respectively. After preparing the samples and activating the reactor, sampling of the solution inside the reactor was done at different time intervals. In addition, sampling was performed from the Kerman hospital wastewater treatment plant and samples transferred at 4 ^°^C for experiments. In the next step, the physicochemical characteristics of the wastewater entering the hospital treatment plant were examined.

### 2.2 Sono-Fenton-like experiments

This study was an experimental research performed in a glass plexiglass container on a laboratory batch sonochemical scale. The used pilot (Batch Reactor) included a cylindrical sonochemical reaction container made of steel with a volume of 1 L. An ultrasound device (DUMAN-120) was used to generate ultrasound waves. In all stages of the Sono-Fenton-like process, a magnetic stirrer with a speed of 1000 rpm at 40 kHz was used to mix samples. Besides, by using HCl 0.1 M and NaOH 0.1 *N*, pH was adjusted. The studied samples were synthetic wastewater made in the laboratory with different concentrations of metronidazole. Amounts of the studied parameters were selected according to similar studies. The parameters of the process including the pH [[3], [4], [5], [6], [7], [8], [9], [10], [11]], metronidazole concentration (25–100 mg/L), reaction time (5–90 min), nanocomposite dosage (0.1–1 g/L), and amount of H_2_O_2_ (0.2–2 mM) were examined and optimized [42,51]. After optimizing the sono-fenton-like process conditions on the synthetic samples, the process has been carried out on the real sample. The real sample was provided of the wastewater treatment plant which is located on the Kerman University of Medical Sciences campus and its physicochemical properties was investigated. Then, the removal efficiency was evaluated on the real sample under the optimal conditions which are achieved from the experiment on the synthetic samples.

### 2.3 Synthesis of Fe_3_O_4_@HZSM-5 magnetic nanocomposite

At first, the iron chloride (II) and iron chloride (III) salts were dissolved (1:2) in 100 mL of double-distilled water. Then, the obtained solution was deoxygenated in the presence of nitrogen gas for 20 min. After that, HZSM-5 zeolite (1 g) was added. In the next step, at 60 °C, ammonia was added dropwise to the reaction plate until black sediment was obtained. The achieved sediment was separated by a magnet and was washed several times with distilled water to neutralize it. Finally, Fe_3_O_4_@HZSM-5 magnetic nanocomposite was dried in an oven at 60 °C for 24 h.

### 2.4 Characterization techniques of Fe_3_O_4_@HZSM-5 magnetic nanocomposite

To characterize the specimens FESEM-EDS-Mapping (FE-SEM TESCAN MIRA3) was used. XRD using Philips X-Pert device (the Netherlands) was employed to realize the cobalt ferrite crystalline structure present in the magnetic nano heterogeneous catalyst. By using VSM (Lake Shore Cryotronics-7404), the Fe_3_O_4_@HZSM-5 magnetic properties were characterized at room temperature. BET method with micrometric model 021LN2 transfer device was used to evaluate the porosity of the magnetic nano heterogeneous catalyst surface area. In order to evaluate the leaching of Fe_3_O_4_@HZSM-5 magnetic nanocomposite, the concentration of Fe (248.3 nm) was measured by using an Atomic Absorption Spectrophotometer (AAS, CTA-3000) in the aqueous media after the adsorption process.

The concentration of metronidazole was analyzed by HPLC equipped with a reverse-phase column (Waters 5 μm ODS2 C18, 250 6 4.6 mm) and an ultraviolet detector. In addition, the acetonitrile/oxalic acid mobile phase at 395 nm was used. In addition, the injection volume and contact time of metronidazole were 20 μL and 6.3 min, respectively. In order to measure the residual concentration of metronidazole in samples below the detection range of the spectrophotometer, high-performance liquid chromatography was applied [52]. After completing the experiments, by using the obtained results, the optimal amounts of the studied parameters were calculated. In this research, each experiment was repeated three times based on the one factor at a time method (OFAT), and finally, the averages of the achieved results were reported. The removal efficiency is calculated according to Eq. (3).

(3) $$\%R=\frac{C_{0}-C_{t}}{C_{0}}\times 100$$

Where C_0_ is the contaminant input concentration (antibiotic) and C_t_ is the concentration of output contaminant.

## 3 Results and discussion

### 3.1 Characterization of synthesized Fe_3_O_4_@HZSM-5 magnetite nanocomposite

#### 3.1.1 FESEM, EDS, mapping, and line-scan of Fe_3_O_4_@HZSM-5

**Fig. 1:**
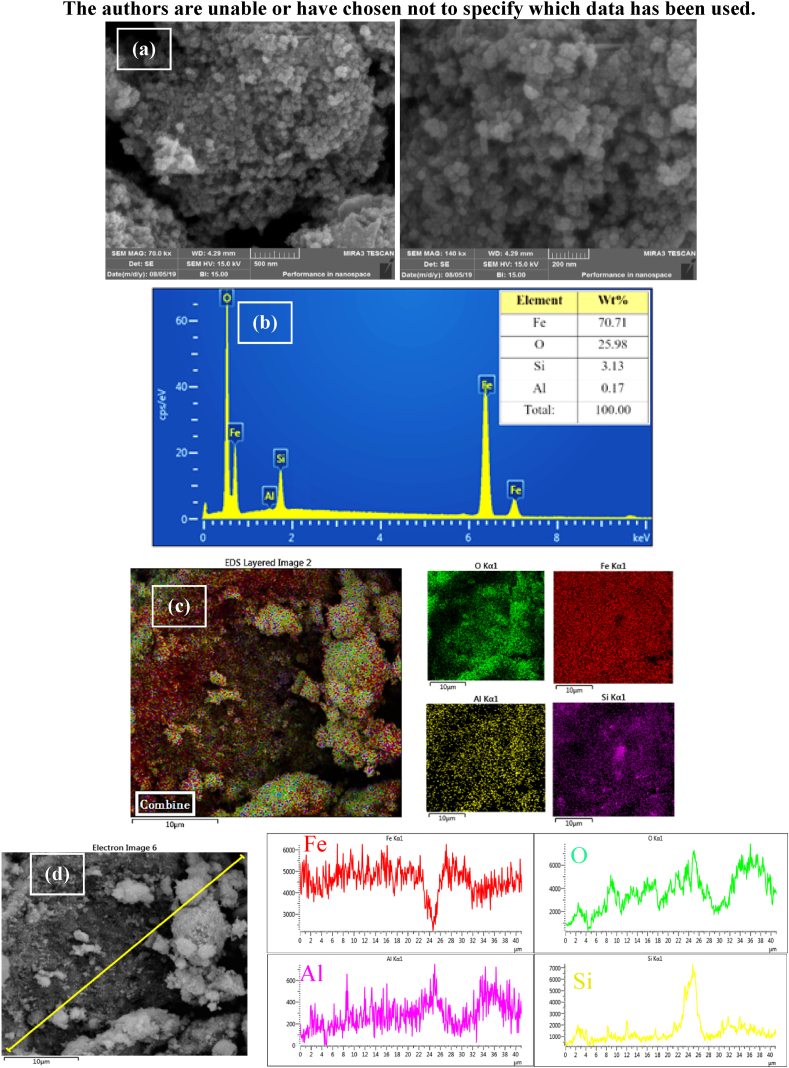
The FESEM images (a), EDS patterns (b), elemental mapping images (c), and line-scan (d) of Fe_3_O_4_@HZSM-5 magnetic nano heterogenous catalyst.

FESEM analysis was used to evaluate the Fe_3_O_4_@HZSM-5 surface morphology and the obtained result is shown in Fig. 1a. Fe_3_O_4_@HZSM-5 arranged as *pseudo*-spherical magnetic nanocomposite with uniformly and loosely aggregated form. The Fe_3_O_4_@HZSM-5 average particle size was obtained at 27 nm. EDS analysis was used to measure the purity and chemical structure of Fe_3_O_4_@HZSM-5 magnetic nano heterogeneous catalyst (Fig. 1b). The achieved results are 25.98% O, 70.71% Fe, 3.13% Si, and 0.17% Al that are matching with the expected values. To investigate Fe_3_O_4_@HZSM-5 elements distribution, Mapping analysis was used. According to the achieved results (Fig. 1c) Al, Si, Fe, and O had a homogeneous distribution that shows the Fe_3_O_4_@HZSM-5 high uniformity. Besides, to study the concentration changes of elements between different areas of the Fe_3_O_4_@HZSM-5, the line-scan analysis was used which approved the Mapping analysis (Fig. 1d).

#### 3.1.2 Magnetic properties of Fe_3_O_4_@HZSM-5 magnetic nanocomposite

**Fig. 2:**
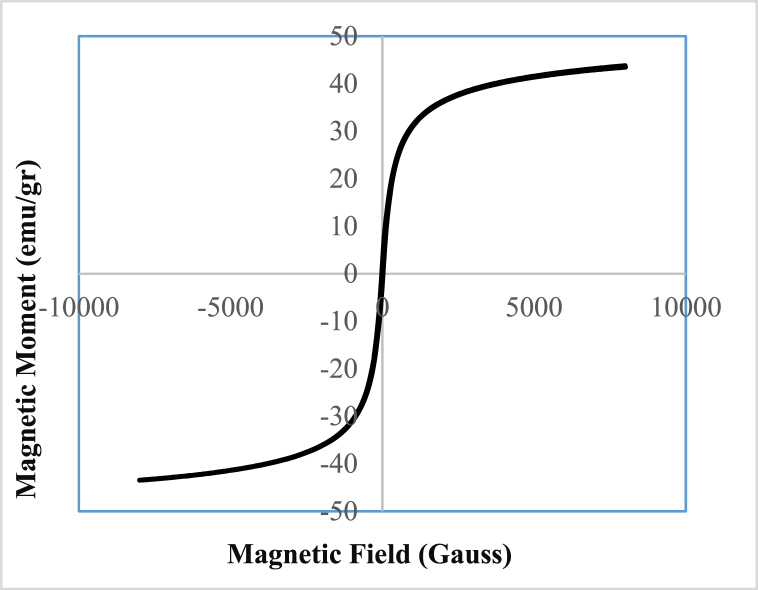
VSM of Fe_3_O_4_@HZSM-5 magnetic nano heterogenous catalyst.

Based on Fig. 2, The Fe_3_O_4_@HZSM-5 remnant magnetization (Mr), coercive force (Hc), and saturation magnetization (Ms) values were obtained at 4.06 emu g^−1^, 50 Oe, and 43.72 emu g^−1^, respectively which indicates the Fe_3_O_4_@HZSM-5 high magnetic strength. The Fe_3_O_4_@HZSM-5 high magnetic property has a helpful role in quickly separating the magnetic nano heterogeneous catalyst from the reaction medium by an external magnet in the reuse and recovery stages.

#### 3.1.3 The crystalline phase properties of Fe_3_O_4_@HZSM-5 magnetic nanocomposite

**Fig. 3:**
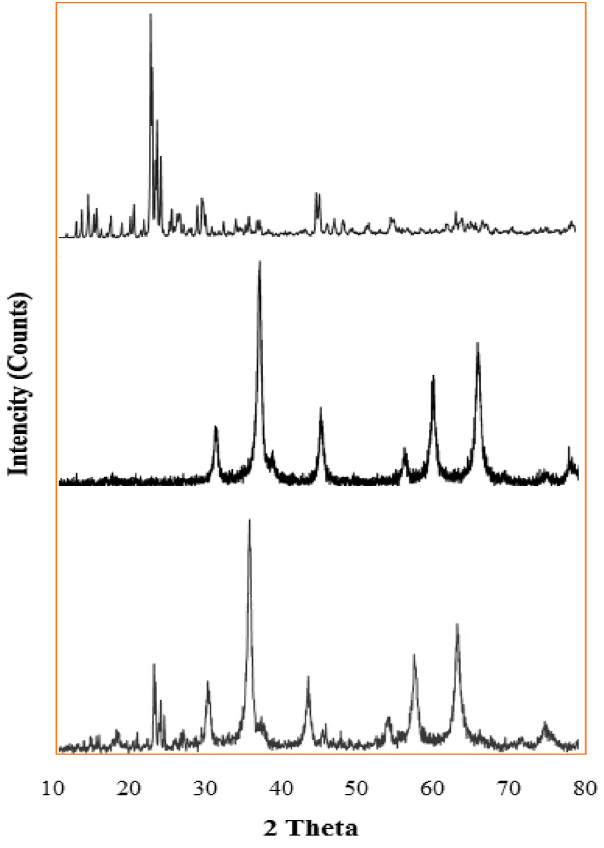
The XRD patterns of HZSM-5 (a), Fe_3_O_4_ (b), and Fe_3_O_4_@HZSM-5 (c) magnetic nano heterogenous catalyst.

HZSM-5, Fe_3_O_4_, and Fe_3_O_4_@HZSM-5 XRD patterns were prepared and compared with each other (Fig. 3). The results showed that the values which are seen in 2*θ* = 30.26^○^, 35.70^○^, 43.35^○^, 53.74^○^, 57.29^○^, and 62.81^○^ belong to Fe_3_O_4_ [53,54] and 23.15^○^ and 24.06^○^ peaks are related to HZSM-5 [55]. The Fe_3_O_4_@HZSM-5 magnetic nanocomposite XRD results in the range from 10 to 80° (2θ = 10^○^- 80^○^) are demonstrated in Fig. 3. The crystalline structure in the spinel shape was consistent with the Joint Committee on Powder Diffraction Standards (JCPDS 98-001-7122) that showed by the sharp diffractions in regions 23.15^○^, 24.06^○^, 30.26^○^, 35.70^○^, 43.35^○^, 53.74^○^, 57.29^○^, and 62.81^○^. The crystalline structure of Fe_3_O_4_ with complete crystallization conserved the crystalline structure of Fe_3_O_4_@HZSM-5 after being composite with zeolite. This phenomenon was demonstrated by the sharp and strong peaks' presence and the comparison of peak locations with reference data. The average value of Fe_3_O_4_@HZSM-5 crystallite size was achieved at 8.67 nm from the Scherrer equation (Eq. (4)) [56].

(4) $$D=\frac{0.9\lambda }{\beta \;\cos\;\theta }$$

#### 3.1.4 The specific surface area of Fe_3_O_4_@HZSM-5 magnetic nanocomposite

**Fig. 4:**
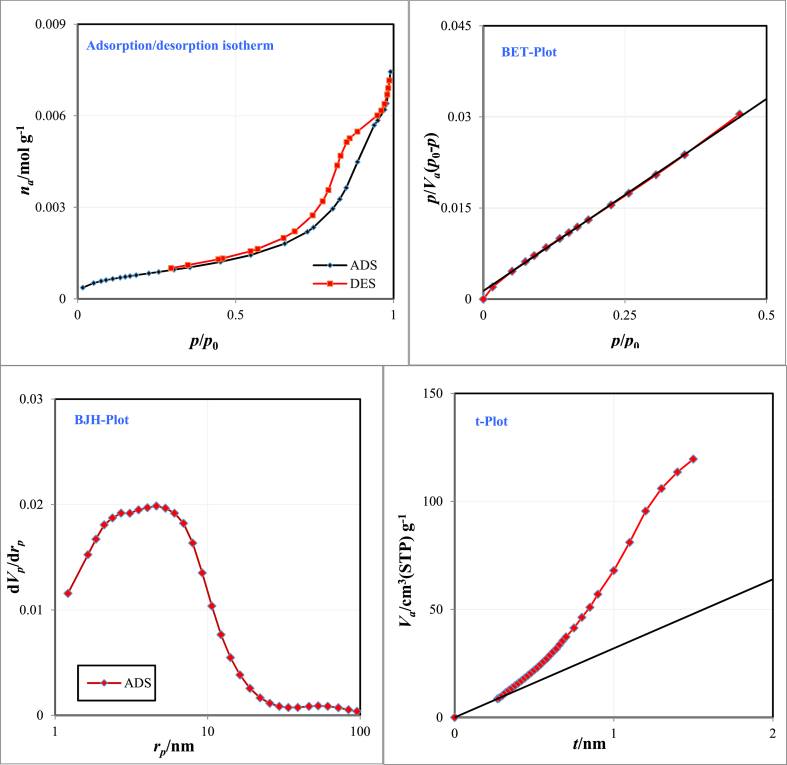
Adsorption/desorption isotherm (a), BET surface area (b), t-Plot (c), and BJH surface area (d) of Fe_3_O_4_@HZSM-5 magnetic nano heterogenous catalyst.

Fig. 4 indicates adsorption/desorption isotherm, BET-BJH specific surface area, and t-plot of the Fe_3_O_4_@HZSM-5 magnetic nanocomposite (Fig. 4a–d). In accordance with the BET plot, the magnetic nano heterogeneous catalyst mean pore diameter, specific surface area, and total pore volume (p/p_0_ = 0.99) were obtained at 15.22 nm, 67.387 m^2^/g, and 0.2564 cm^3^/g, respectively. According to the IUPAC explanation, pore materials, according to their sizes are arranged into three groups: microporous (smaller than 2 nm), mesoporous (between 2 and 50 nm), and macroporous (larger than 50 nm) [57]. Fe_3_O_4_@HZSM-5 is classified as a mesoporous material.

### 3.2 Effects of parameters on the degradation of metronidazole in the Sono-Fenton-like process

#### 3.2.1 Effect of oxidant concentration

**Fig. 5:**
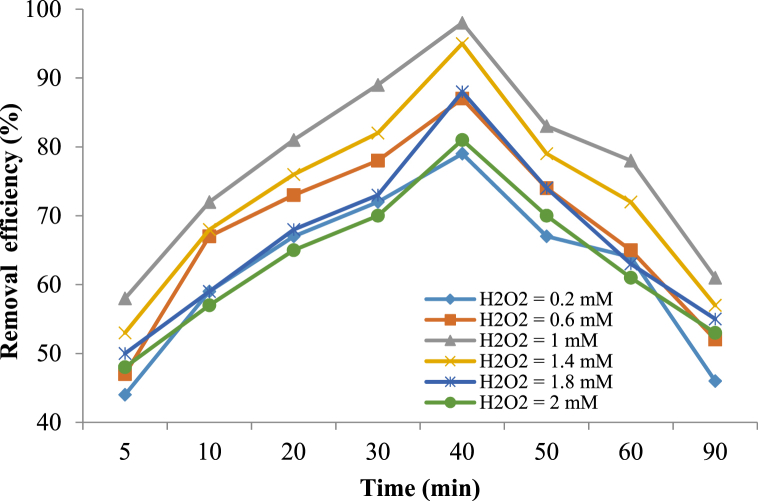
Effect of oxidant concentration on the metronidazole removal efficiency (catalyst dose 0.4 g/L, metronidazole initial concentration 25 mg/L, pH 7 and reaction time 40 min).

Hydrogen peroxide is the source of hydroxyl radical production and it can play an important role in the oxidation process [58]. Nevertheless, its excessive use reduces the removal efficiency and increases the process costs. Fig. 5 shows the effect of H_2_O_2_ on the removal efficiency at pH = 7, metronidazole initial concentration of 25 mg/L, nanocomposite dose 0.4 g/L, and reaction time 40 min.

By increasing the amount of H_2_O_2_ from 0.2 mM to 1 mM, the metronidazole removal efficiency increased from 79% to 98%, which showed the great effect of the amount of H_2_O_2_ in the solution. Increasing the H_2_O_2_ concentration from 1 mM to 2 mM reduced the removal efficiency from 98% to 81%. According to these results, the hydrogen peroxide optimal concentration was obtained at 1 Mm. Increasing concentration of the hydrogen peroxide leads to increasing the hydroxyl radical production and increasing the removal efficiency. According to the following equations, in the high concentrations of H_2_O_2_, excessive amounts of H_2_O_2_ in the medium can play the role of radical scavenger (radical scooper) that reduces the active hydroxyl radical species during the oxidation process. These H_2_O_2_ excess values can react with the hydroxyl radicals (^•^OH) and produce hydroperoxyl radicals (HO^•^_2_), which have lower oxidation potential than ^•^OH radicals [59](Eqs (5), (6), (7)).

(5) H_2_O_2_+^•^OH → H_2_O + HO^•^_2_

(6) HO^•^_2_+^•^OH → H_2_O + O_2_

(7) ^•^OH+ ^•^OH → H_2_O_2_

In addition, in the H_2_O_2_ higher concentrations, this compound can be adsorbed on the Fe_3_O_4_@HZSM-5 magnetite nanocomposite surface and limit the reactant concentration of metronidazole.

Hassani et al. (2018) reported that the optimal AO_7_ removal by CoFe_2_O_4_-rGO nanocomposite was achieved at H_2_O_2_ = 2 Mm, but with increasing H_2_O_2_, the removal efficiency decreased [59]. Also, other research conducted by Xu et al. (2012) showed that the degradation of 2,4-dichlorophenol by using Fe_3_O_4_ magnetic nanoparticles increased by increasing H_2_O_2_ concentration until 12 Mm and the higher concentration of H_2_O_2_ causes lower 2,4-dichlorophenol removal efficiency [60]. These results were consistent with the results of the current research.

#### 3.2.2 Effect of Fe_3_O_4_@HZSM-5 dosage

**Fig. 6:**
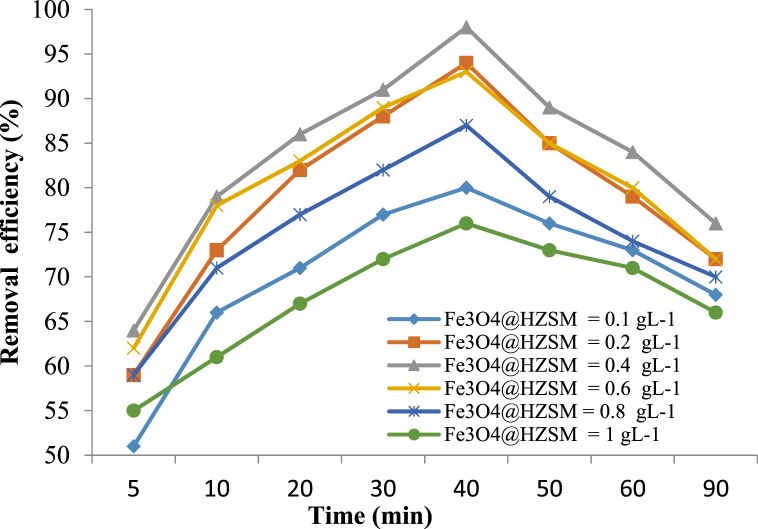
The effect of Fe_3_O_4_@HZSM-5 dosage on the metronidazole removal efficiency (H_2_O_2_ concentration 1 mM, metronidazole initial concentration 25 mg/L, pH 7 and reaction time 40 min).

The results of the changes in the Fe_3_O_4_@HZSM-5 magnetite nanocomposite dosage are shown in Fig. 6. With increasing the amount of Fe_3_O_4_@HZSM-5 magnetite nanocomposite from 0.1 to 0.4 g/L, the removal efficiency was increased from 80% to 98% in 40 min, and the removal efficiency was decreased. Also, with increasing the amount of the Fe_3_O_4_@HZSM-5 magnetite nanocomposite from 0.4 to 1 g/L removal efficiency decreased to 76%. Fe_3_O_4_@HZSM-5 magnetite nanocomposite, as a peroxidase-like catalyst, could decompose H_2_O_2_ into ^•^OH radicals swiftly. Therefore, the amount of Fe_3_O_4_@HZSM-5 was an important factor in the Sono-Fenton-like process that could significantly enhance the metronidazole degradation. The metronidazole degradation efficiency decreased, probably because with decreasing the amount of the Fe_3_O_4_@HZSM-5 magnetite nanocomposite, the catalyst surface area to adsorb H_2_O_2_ was reduced too. The removal efficiency decreased with increasing the amount of catalyst from 0.4 to 1 g/L. This increase in the catalyst dosage can act as a scavenger and reduce the process removal efficiency. On the other hand, when the nanocomposite dosage reaches above the saturation level, the energy of the ultrasonic waves is not sufficient to disperse the catalyst. Moreover, high amounts of catalysts can lead to condensation and accumulation of the catalyst nanoparticles and reduce their active surface, so hydroxyl radical production decreases. Therefore, the removal efficiency decreases with increasing catalyst dosage. Also, after 40 min, the removal efficiency of the process decreased. This can be due to the intermediate compounds that are produced in the solution during the process and occupy the active sites of the catalyst surface and reduce the efficiency of the process.

Zhang et al. (2020) and Forouzesh et al. (2019) studied degradation of chloramphenicol and metronidazole, respectively. They concluded that increasing catalyst dosage can lead to removal efficiency increase [61,62]. The results of these studies are consistent with the current study.

#### 3.2.3 Effect of initial metronidazole concentration

**Fig. 7:**
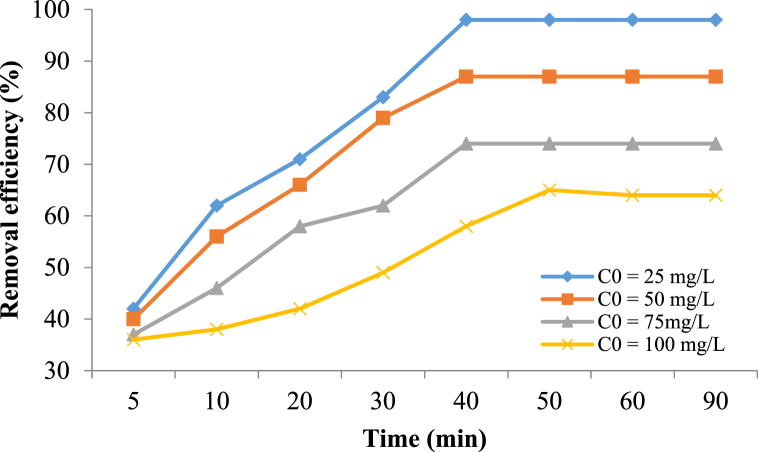
The effect of initial metronidazole concentration (Catalyst dose 0.4 g/L, H_2_O_2_ concentration 1 mM, pH 7 and reaction time 40 min).

At this stage, the effect of metronidazole concentration was investigated and the obtained results are presented in Fig. 7. The results showed a decrease in the removal efficiency of the combined process with increasing the initial metronidazole concentration. At metronidazole concentrations of 25 mg/L and 100 mg/L, the removal efficiencies decreased from 98% to 58%, respectively.

The results indicated that the removal efficiency decreased with increasing the metronidazole concentration. The reason for this decrease could be that with increasing the metronidazole concentration in the solution, more molecules of the pollutant can block the Fe_3_O_4_@HZSM-5 nanocomposite active sites of the catalyst surface and cause a reduction in the ^•^OH radical production, and following that the removal efficiency decreases. In addition, the catalyst adsorbs the pollutant molecules on its surface, which prevents the catalyst from absorbing the energy developed by the acoustic cavitation. Therefore, the production of hydroxyl radicals is reduced. In addition, at high amounts of metronidazole, pollutant and intermediate molecules that are produced during the Sono-Fenton-like oxidation process compete with each other to react with the hydroxyl radicals and cause the removal efficiency reduction. In addition, increasing the metronidazole concentration causes more oxidant consumption and increases the decomposition process time.

The achieved results of the muthirulan et al. (2013) [63] study showed that at higher initial concentrations of dye, the heterogeneous sonocatalytic process efficiency decreases. Malakootian et al. (2019) studied tetracycline antibiotics removal using ultrasound/Fe_3_O_4_ nanoparticles/persulfate. The results demonstrated with increasing the pollutant concentration the removal efficiency decreased [64] which confirms the present study results.

#### 3.2.4 Effect of pH

**Fig. 8:**
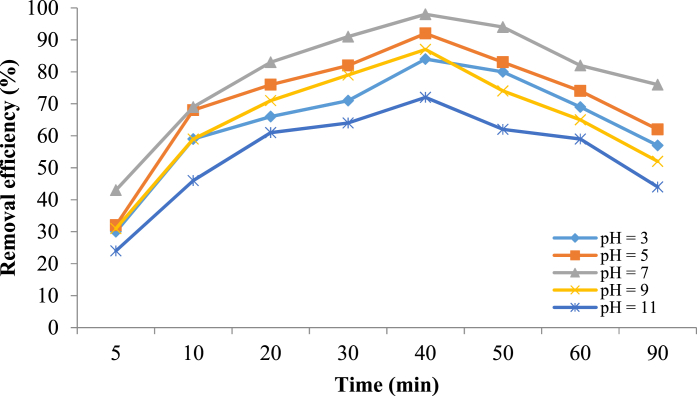
The effect of pH on the metronidazole removal efficiency (Catalyst dose 0.4 g/L, H_2_O_2_ concentration 1 mM, metronidazole initial concentration 25 mg/L and reaction time 40 min).

pH is one of the most important and influential parameters in the Sono-Fenton-like process which can control the ^•^OH production amount and, the ferrous ion concentration. The effect of pH on the metronidazole removal efficiency is shown in Fig. 8. In order to investigate the effect of pH in the afore-mentioned process, pH values in the range of 3–11, with metronidazole initial concentration 25 mg/L, reaction time 40 min, and catalyst dosage 0.4 g/L were examined. The achieved results demonstrated that the removal efficiency of metronidazole increases with increasing pH. The highest efficiency was obtained at 98% at pH 7. Nevertheless, with increasing pH, the removal efficiency decreased. In general, the removal efficiency in the acidic and neutral conditions was better than the alkaline pHs. Increasing the pH value from 3 to 11 in the solution can lead to decreasing the oxidation potential of ^•^OH/H_2_O redox pair from 2.59 to 1.65 V and increasing the standard hydrogen electrode (SHE) [65]. Overall, it can be concluded that the oxidation potential of ^•^OH radicals in acidic solution is higher than in the alkaline solution. Additionally, the Fe_3_O_4_@HZSM-5 magnetite nanocomposite has zero point of charge (pH_zpc_) (Fig. 8). In the solutions with a pH lower than pH_zpc_, the catalyst surface is protonated, and on the contrary, in the solutions, with a pH higher than pH_zpc_ the catalyst surface will be deprotonated [66]. Thus, metronidazole can be adsorbed better on the Fe_3_O_4_@HZSM-5 surface in the acidic medium. In addition, in the acidic pHs, the dissolved iron concentration increases. This increase can lead to an increase in the production of hydroxyl radicals in the heterogeneous Fenton process. In addition, at high pHs, H_2_O_2_ molecules decompose into oxygen and water. Consequently, because of reducing ^•^OH amount, the removal efficiency is decreased [67].

Hu et al. (2011) conducted a study about metronidazole degradation by using Fe_3_O_4_ magnetic nanoparticles and reported that the highest removal efficiency achieved at pH 3 and by increasing pH the degradation rate decreased rapidly [67].

### 3.3 Synergistic effect between the metronidazole sonochemical and catalytic degradation

**Fig. 9:**
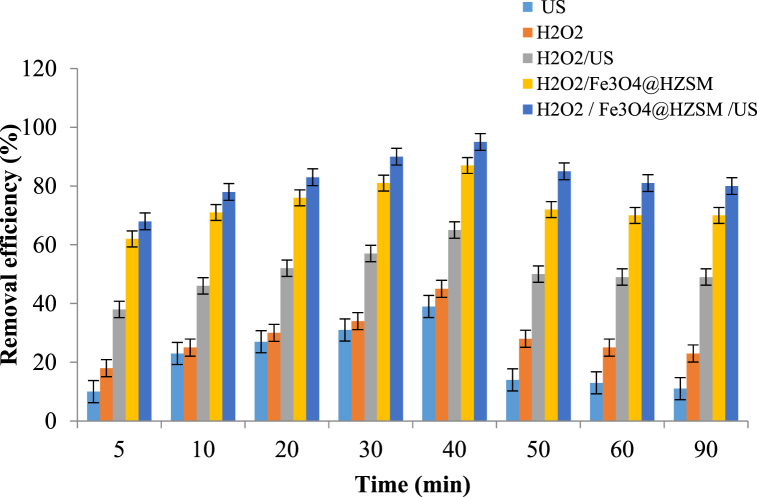
Comparison of removal efficiency in different modes (catalyst dose 0.4 g/L, H_2_O_2_ concentration 1 mM, metronidazole initial concentration 25 mg/L, pH 7 and reaction time 40 min).

The metronidazole removal efficiency was evaluated in different conditions. The obtained results showed (Fig. 9) that each condition did not have good removal efficiency lonely but in the integrated process (Fe_3_O_4_@HZSM-5/H_2_O_2_/US), a suitable metronidazole removal efficiency was observed. In addition, by using an iron-free catalyst (HZSM-5/H_2_O_2_/US) metronidazole removal efficiency was assessed. Reduction in the iron potential showed that the iron-containing catalyst has more oxidation power than the iron-free.

Oxidation time is another parameter that has an effect on the process efficiency. In order to determine the best time and its effect on the Sono-Fenton-like process, the process efficiency was evaluated from 5 to 90 min. Over time, the metronidazole removal rate increased. Therefore, the maximum removal at 40 min was obtained 95% and after that, the removal rate remained constant. Increasing the removal efficiency with increasing the oxidation time can be due to producing more active hydroxyl radicals and having sufficient opportunity to react with metronidazole. With increasing process time from 40 to 90 min, no increase in the removal efficiency was observed and this might be due to the formation of carbonate and bicarbonate ions in the process media, which can reduce the effect of hydroxyl radicals [68].

### 3.4 Kinetic study of metronidazole degradation

*Pseudo*-first-order kinetic (Eq. (8)) and Langmuir-Hinshelwood models (Eq. (9)) were evaluated in order to metronidazole degradation kinetics investigation. Langmuir-Hinshelwood is the commonplace kinetic model to explain heterogeneous catalytic processes. In this model, the pollutant adsorption on the catalyst active sites is evaluated [66].

(Eq. 8) $$\mathrm{Ln}\frac{C_{t}}{C_{0}}=-K_{obs}t$$

Where C_0_ and C_t_ (mg L^−1^) represent the metronidazole initial concentration and after reaction time, respectively and K_obs_ is the reaction rate constant (min^−1^).

(Eq. 9) $$\frac{1}{K_{obs}}=\frac{1}{K_{C}K_{L-H}}+\frac{C_{0}}{K_{C}}$$

Where K_c_ is the surface reaction rate constant (mg L^−1^ min^−1^) and K_L-H_ is the adsorption equilibrium constant (L mg^−1^) [66].

The amount of K_obs_ was achieved by scheming Ln (C_t_) versus time in the different concentrations that are shown in Table 1.

**Table 1:** *Pseudo*-first-order kinetic parameters of MNZ degradation.

Entry	C_0_	R^2^	K_obs_	Line Eq.
1	25	0.9596	0.0462	y = −0.0462x + 2.814
2	50	0.9605	0.036	y = −0.036x + 3.499
3	75	0.9563	0.0204	y = −0.0204x + 3.9065
4	100	0.9552	0.0134	y = −0.0134x + 4.2693

**Fig. 10:**
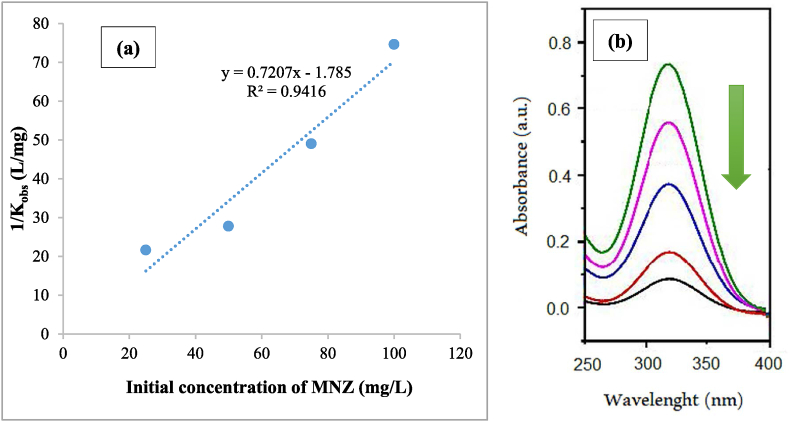
Langmuir-Hinshelwood kinetic curve (a) and variation in UV–vis spectra of metronidazole with reaction time (catalyst dose 0.4 g/L, H_2_O_2_ concentration 1 mM, metronidazole initial concentration 25 mg/L, and pH 7) (b).

After that, a linear equation was obtained by plotting the curve K_obs_^−1^ against the metronidazole initial concentration and by using it K_c_ and K_L-H_ values were calculated (Fig. 10 a). Based on the achieved results the amounts of K_L-H_ and K_c_ were 0.40 L mg^−1^ and 1.38 mg L^−1^ min^−1^ respectively, and it was shown that degradation of the metronidazole follows Langmuir-Hinshelwood kinetics and *pseudo*-first-order. Nasiri et al. carried out a study on ciprofloxacin removal and reported that the ciprofloxacin degradation follows *pseudo*-first-order and Langmuir-Hinshelwood kinetics [69]. The changes in spectra intensity of metronidazole removal under optimal conditions (catalyst dose 0.4 g/L, H_2_O_2_ concentration 1 mM, initial concentration 25 mg/L, pH 7, and reaction time 40 min) and different times are demonstrated in Fig. 10 b. The metronidazole absorption peak was achieved at *λ*_max_: 321.5 nm. With decreasing the metronidazole concentration, the absorption intensity was decreased too.

### 3.5 Reusability and chemical stability of Fe_3_O_4_@HZSM-5

**Fig. 11:**
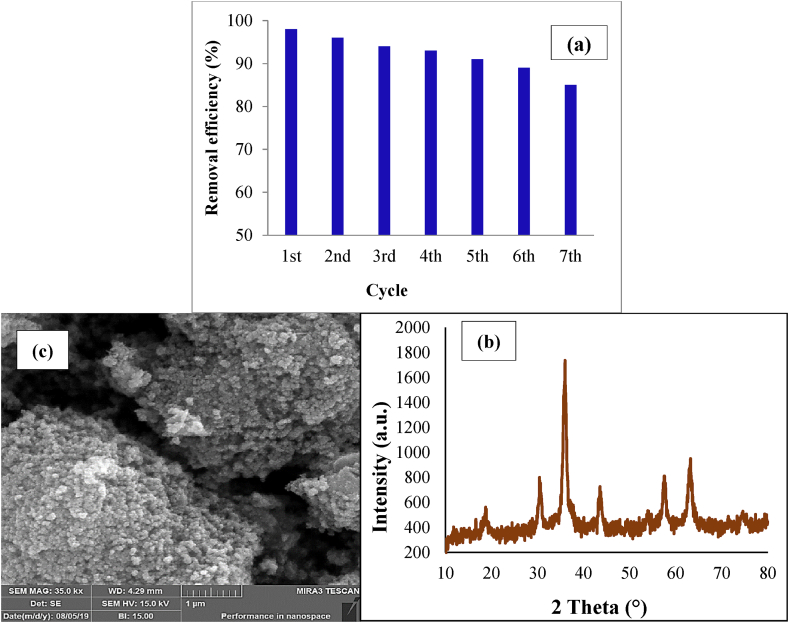
Regeneration (catalyst dose 0.4 g/L, H_2_O_2_ concentration 1 mM, metronidazole initial concentration 25 mg/L, pH 7 and reaction time 40 min) (a), FESEM image (b) and XRD analysis (c) of Fe_3_O_4_@HZSM-5 magnetic nano-heterogeneous catalyst after seven recycling cycles.

Due to economic and environmental reasons and their importance in the advanced oxidation processes (AOPs), the reusability of Fe_3_O_4_@HZSM-5 was evaluated. The obtained results are shown in Fig. 11a. At first, Fe_3_O_4_@HZSM-5 was separated from the solution by using a magnet and then washed with EtOH/H_2_O for seven cycles. The results showed that the metronidazole removal efficiency decreased to 98% after the first cycle. In addition, occupation of catalyst-active sites with metronidazole and the reduction amount of Fe_3_O_4_@HZSM-5 during the recycling process can be the reason for the significant reduction in the removal efficiency after the 7th cycle reaching 85%. The chemical stability of Fe_3_O_4_@HZSM-5 was determined after seven regeneration cycles. In order to reach this aim, the concentration of Fe ions was measured (248.3 nm wavelength) by using an Atomic Absorption Spectrophotometer (AAS, CTA-3000). The concentration of Fe ions was achieved at 0.6 mg/L, which illustrates the appropriate chemical stability of Fe_3_O_4_@HZSM-5. In addition, FESEM and XRD analyses of the Fe_3_O_4_@HZSM-5 were performed. According to the obtained results are shown in Fig. 11b and c, no significant changes were observed in the position of 2 Theta and intensity of picks and the Fe_3_O_4_@HZSM-5 morphology. Nevertheless, a reduction slightly was done in the amount of peak diffraction intensity, and the catalyst crystal structure was preserved after seven recycling cycles (Fig. 11b). Consequently, Fe_3_O_4_@HZSM-5 has good chemical stability and is the easily recoverable catalyst.

### 3.6 Proposed mechanism of metronidazole degradation in the Sono-Fenton-like process

**Fig. 12:**
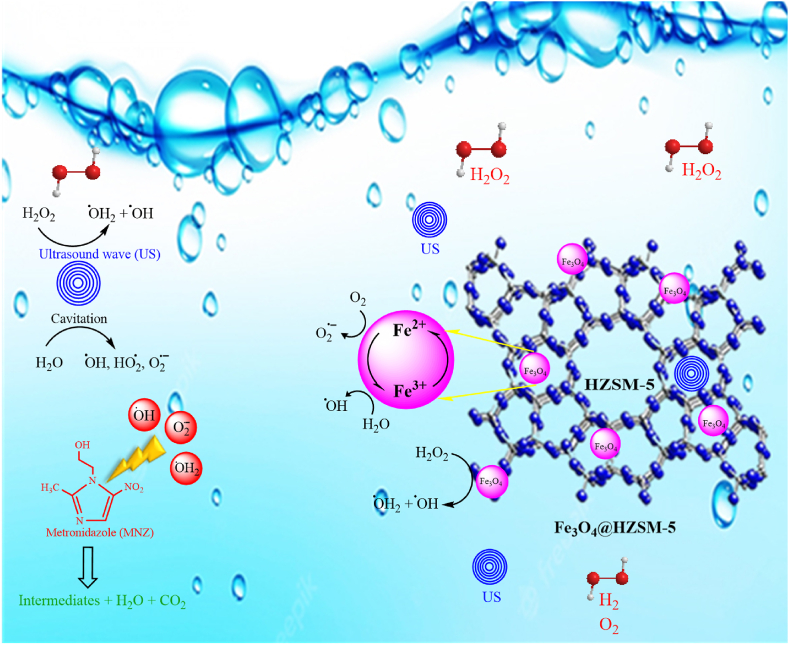
Proposed mechanism of metronidazole degradation.

The proposed mechanism of metronidazole degradation using the heterogeneous magnetic catalyst Fe_3_O_4_@HZSM-5 during the Heterogeneous Sono-Fenton like process is shown in Fig. 12. The basis of metronidazole degradation during the Heterogeneous Sono-Fenton like process is based on the production of free radicals of hydroxyl (^●^OH), superoxide (O_2_^−●^) and hydroperoxyl (HO^●^_2_) in the reaction medium. In the metronidazole removal mechanism, hydrogen peroxide decomposition is performed on the catalyst surface and in the liquid phase as a reaction substrate. After the hydrogen peroxide degradation, the hydroxyl and superoxide radicals were formed on the catalyst surface and released into the aqueous media. In addition, Fe^2+^/Fe^3+^ ions on the catalyst surface reacted with hydrogen peroxide and produced the hydroxyl and hydroperoxyl radicals with a lower oxidation potential than hydroxyl radicals. Following the reaction of ferrous ions (Fe^2+^) with hydrogen peroxide, ferric ions (Fe^3+^) were produced. Following the reaction of Fe^3+^ with H_2_O_2_, Fe^2+^ ions were regenerated and reduced and hydroperoxyl radicals were produced. In addition, ultrasonic waves caused the production of hydroxyl, superoxide, and hydroperoxyl radicals during the cavitation of water molecules. In addition, the hydrogen peroxide molecules generated the hydroxyl and hydroperoxyl radicals near ultrasonic waves. Eventually, the metronidazole molecules were converted into inorganic compounds by the hydroxyl (^●^OH), superoxide (O_2_^−●^), and hydroperoxyl (HO^●^_2_) free radicals.

### 3.7 Effect of radical scavenger

**Fig. 13:**
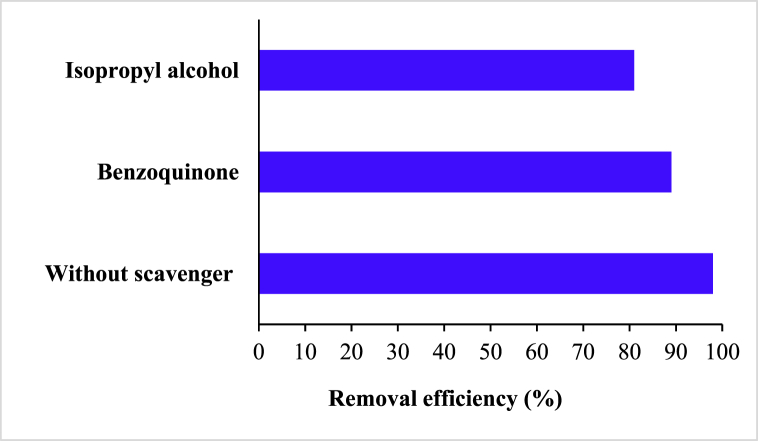
Effect of radical scavengers on the metronidazole degradation (5 mg/L radical scavenger, catalyst dose 0.4 g/L, H_2_O_2_ concentration 1 mM, metronidazole initial concentration 25 mg/L, pH 7 and reaction time 40 min).

To identify active radical species in the metronidazole degradation, scavenging tests were performed in the optimum conditions (catalyst dose 0.4 g/L, H_2_O_2_ concentration 1 mM, metronidazole initial concentration 25 mg/L, pH 7 and reaction time 40 min). For trapping superoxide radicals (^•^O_2_^−^) and hydroxyl radicals (^•^OH) isopropyl alcohol (5 mg/L) and benzoquinone (5 mg/L) were applied, respectively. It can be concluded from obtained results (Fig. 13) that adding radical scavengers to suspensions caused a decrease in the metronidazole removal efficiency.

Metronidazole removal efficiency in the benzoquinone and isopropyl alcohol presence was achieved 89% and 81%, respectively. As can be seen, the metronidazole removal efficiency decreased. Both effective quencher, benzoquinone, and isopropyl alcohol had the lowest and highest noticeable effects on metronidazole degradation, respectively. Therefore, the important role of hydroxyl radicals in the metronidazole degradation was approved in the research, and the presence of both scavengers caused the process efficiency reduction. The previous researches are consistent with the obtained results (67).

### 3.8 Mineralization

In order to report the level of MNZ mineralization by the Sono-Fenton-like oxidation, the amount of TOC removal was evaluated. The removal efficiencies of metronidazole and TOC under the optimal conditions at 40 min were obtained 98% and 81%, respectively, which showed the high process efficiency in degradation and mineralization.

### 3.9 Treatment of real wastewater

As part of this study, the Sono-Fenton-like oxidation result on the metronidazole degradation in real wastewater was evaluated too. At first, a sample was provided of the wastewater treatment plant which is located on the Kerman University of Medical Sciences campus with characteristics COD: 28.2 mg/L, BOD_5_: 10.2 mg/L, TSS: 85 mg/L, TDS: 1200 mg/L, TKN: 2.23 mg/L, Phosphate: 35.30 mg/L, Nitrate (NH_3_): 2.5 mg/L, Sulfate: 141.1 mg/L, pH: 7 and metronidazole 25 mg/L. Then, under the optimal conditions (catalyst dose 0.4 g/L, H_2_O_2_ concentration 1 mM, initial concentration 25 mg/L, pH 7, and reaction time 40 min) achieved from the experiment on the synthetic samples, the removal efficiency was assessed at 83%. The result shows that the Sono-Fenton-like oxidation process has the appropriate efficiency in real wastewater treatment. Because of impurities' presence such as COD, BOD, etc., the metronidazole removal efficiency in real wastewater is lower than the synthetic. Therefore, for removing these impurities, the Sono-Fenton-like oxidation process is used. In other words, interference between cations and anions may act as scavengers and decrease the function of free radicals.

### 3.10 Comparison of metronidazole degradation efficiency in the other AOPs

The Sono-Fenton-like process efficiency in the presence of Fe_3_O_4_@HZSM-5 magnetic nano heterogeneous catalyst is compared with other Fenton-like processes in Table 2.

**Table 2:** Comparison of the performance process *vs* other processes.

No.	Type of process	Catalyst	Pollutant	Initial Conc. (mg/L)	Catalyst dose (g/L)	H_2_O_2_ (mM)	Time (min)	Removal Efficiency (%)			Recovery (%)	Ref.
								Real effluent	Synthetic effluent	TOC		
1	heterogeneous Fenton	Fe_3_O_4_	Ciprofloxacin	10	1.7	12	120	–	89	_	65.7	[70]
2	Fenton-like	D-Fe@Sep	Ofloxacin	10	3	30	120	–	93	85.56	–	[71]
3	Fenton-like	Fe_3_O_4_/PAC	Sulfamethazine	20	1	20	120	–	94.7	80.8	74	[72]
4	Sono-Fenton like	Fe_3_O_4_@ HZSM-5	Metronidazole	25	0.4	1	40	83	98	81	85	Present Study

According to the reported results, the Sono-Fenton-like process compared to the other processes has the highest removal efficiency in synthetic and real samples with a higher concentration of pollutants, a lower dose of catalyst, a lower amount of consuming oxidant, and in a shorter time.

## 4 Conclusion

In summary, the Fe_3_O_4_@HZSM-5 nano-magnetite heterogeneous catalyst was synthesized using co-precipitation method. The synthesized magnetite nanocomposite was characterized with FESEM, EDS, Line Scan, Dot Mapping, XRD, VSM, and BET analysis. The magnetic nanocomposite structural analysis showed that the average particle size of the Fe_3_O_4_@HZSM-5 was obtained 27 nm. The achieved results of EDS are 25.98% O, 70.71% Fe, 3.13% Si, and 0.17% Al that are matching with the expected values. In the Mapping analysis Al, Si, Fe, and O had a homogeneous distribution that shows the Fe_3_O_4_@HZSM-5 high uniformity. Based on VSM, the remnant magnetization (Mr = 4.06 emu g^−1^), coercive force (Hc = 50 Oe), and saturation magnetization (Ms = 43.72 emu g^−1^) were obtained which indicates the Fe_3_O_4_@HZSM-5 high magnetic strength. The sharp and strong peaks demonstrated that the Fe_3_O_4_ crystalline structure with complete crystallization conserved after being composite with zeolite. The average crystallite size was achieved at 8.67 nm. In accordance with the BET analysis, the magnetic nano heterogeneous catalyst mean pore diameter (15.22 nm), specific surface area (67.387 m^2^/g), and total pore volume (0.2564 cm^3^/g) were obtained. Fe_3_O_4_@HZSM-5 is classified as a mesoporous material. Fe_3_O_4_@HZSM-5 nanocatalyst showed high efficiency in the optimal conditions (reaction time 40 min, catalyst dose 0.4 g/L, H_2_O_2_ concentration 1 mM, MNZ initial concentration 25 mg/L, and pH 7) for removing MNZ from the synthetic and real samples with efficiency of 98% and 83%. Besides, by doing the radical scavenger experiments, it was found that the hydroxyl radicals as active radical species played an essential role in the oxidation and degradation of MNZ. After the 7 cycles, the Fe_3_O_4_@HZSM-5 nanocatalyst showed a suitable recovery and reusability in the MNZ removal by 85%. In the future, to modify various spinel metal ferrites and choose suitable magnetic nanomaterials for designing functional magnetic nanocomposites different kinds of minerals can be applied. To remove various organic and inorganic pollutants from contaminated water and wastewater can be used of modified spinel metal ferrites in environmental remediation or magnetic heterogeneous catalysis.

## Author contribution statement

Ghazal Yazdanpanah performed the experiments. Mohammad Reza Heidari analyzed and interpreted the data. Najmeh Amirmahani contributed reagents, materials, analysis tools or data. Alireza Nasiri conceived and designed the experiments. Alireza Nasiri and Ghazal Yazdanpanah wrote the paper.

## Data availability statement

The authors are unable or have chosen not to specify which data has been used.

## Declaration of competing interest

The authors declare that they have no known competing financial interests or personal relationships that could have appeared to influence the work reported in this paper.
