# Erratum to “Heterogeneous Sono-Fenton like catalytic degradation of metronidazole by Fe_3_O_4_@HZSM-5 magnetite nanocomposite” [Heliyon Volume 9, Issue 6, June 2023, Article e16461]

**DOI:** 10.1016/j.heliyon.2025.e43230

**Published:** 2025-03-22

**Authors:** Ghazal Yazdanpanah, Mohammad Reza Heidari, Najmeh Amirmahani, Alireza Nasiri

**Affiliations:** a Environmental Health Engineering Research Center, Kerman University of Medical Sciences, Kerman, Iran; b Environmental Health Engineering, Department of Environmental Health, School of Public Health, Bam University of Medical Sciences, Bam, Iran

In the original published version of this article, the Data Availability Statement is included in Figure 1:

**Figure:**
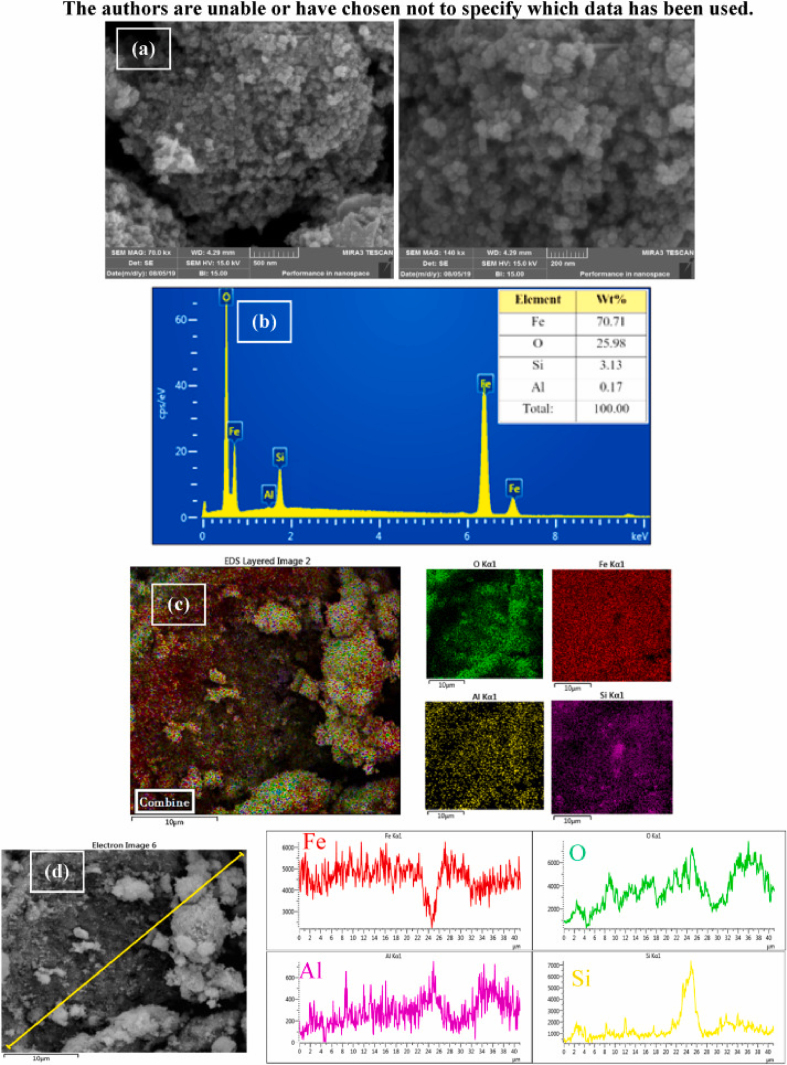

The correct version of Figure 1 can be found below:

**Figure:**
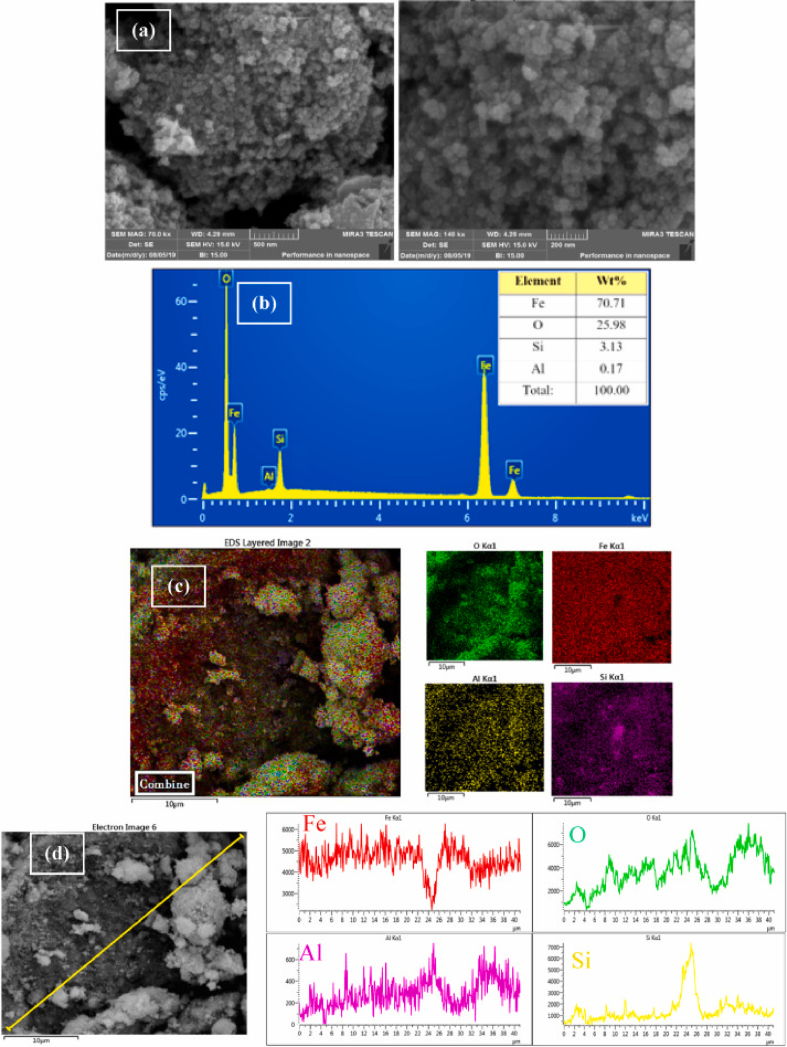

The publishers apologize for the errors.

## Declaration of competing interest

The authors declare that they have no known competing financial interests or personal relationships that could have appeared to influence the work reported in this paper.

